# Controllable Assembly and Application of Janus Smart Nanosheets for Oil Displacement

**DOI:** 10.3389/fchem.2020.00154

**Published:** 2020-03-04

**Authors:** Fang Shi, Jingchun Wu, Yang Zhao, Bo Zhao, Xiangting Kong

**Affiliations:** ^1^Key Laboratory for EOR Technology (Ministry of Education), Northeast Petroleum University, Daqing, China; ^2^Daqing Oil Field Co., Ltd. No. 6 Oil Production Plant Test Brigade, Daqing, China; ^3^Hulunbuir Branch of Daqing Oilfield Co., Ltd., Daqing, China

**Keywords:** oil displacement agent, smart nanosheet, asymmetric Janus particles, ultra-low interfacial tension, dynamic emulsification equilibrium

## Abstract

Based on the development status of low permeability reservoirs, an intelligent nano-flooding agent is needed to improve the displacement efficiency of reservoirs. Janus particles have the characteristics of small size and strong interfacial activity, and the solid surfactant of Janus particles has attracted more and more attention of petroleum researchers. Janus smart nanosheets were developed by Pickering emulsion preparation. Controllable assemblies of Janus smart nanosheets were formed by adjusting the preparation ratio. The structure and properties of smart nanosheets were characterized by Fourier transform infrared spectroscopy, scanning electron microscopy and interfacial tensiometer. The nanosheets have hydrophilic and hydrophilic properties. The particle size of silica nanoparticle is 10 nm. After surface modification and high shear stress treatment, nanosheet was formed. The thickness of nanosheet dispersed in aqueous solution was 30.2 nm. Experimental results show that at a given temperature, the Janus nanosheet system with low concentration can achieve ultra-low interfacial tension of 10^−4^ mN /m, and the nanosheets have good emulsifying ability. The results provide basic insights into the bio-assembly behavior and emulsifying properties of Janus smart nanosheets, and further prove their potential for enhancing oil recovery.

## Introduction

With the increasingly harsh reservoir conditions, scientific research problems such as higher water content, high salinity, ultra-low permeability, and dispersed distribution of remaining oil in reservoir gradually emerge (Finkle et al., 1923). Conventional oil displacement system has some problems, such as poor temperature resistance, salt resistance, shear resistance, and easy degradation, which lead to the failure of oil displacement agent. Based on the properties of nanoparticles, these problems can be effectively solved. Nano-oil displacement agent usually uses silica as basic material. Negatively charged silicon dioxide has strong anti-adsorption ability in reservoirs with the same negative charge (Aveyard et al., 2003; Roh et al., 2005; Glaser et al., 2006). Organic combination of silicon dioxide and conventional displacement agents such as polymers can form electrostatic assemblies and enhance the three important properties of displacement system: temperature resistance, salt resistance, and shear resistance.

Since the 1990s, functional design of Janus materials has attracted the attention of researchers from all walks of life. As a new material that can be used for composition and structure micro-control, Janus material has different properties and wide application prospects by virtue of its unique surface chemical partition and morphology structure, and has become a new breakthrough point in the field of materials. In the past 20 years, many novel methods have been developed to prepare Janus materials with controllable size, shape, microstructure and chemical composition. No matter what the approach is, it needs to rely on one point. That is, to break the symmetrical structure of the original particle shape or composition and to get a multi-functional assembly.

At present, the development of nano-oil displacement agent is mainly through the structural modification of silica to achieve the purpose of functional modification (Binks and Clint, 2002). Single modified silica nanoparticles have strong hydrophilicity or oil affinity. Although single modified silica nanoparticles can solve different types of displacement problems, they all have limitations. The common chemical modification schemes are basically uniform modification, mainly emulsion polymerization, suspension polymerization and so on. The methods of Janus preparation mainly include interface protection modification, phase separation and block copolymer self-assembly. Ling et al. (2009) prepared Janus particles by embedding SiO_2_ particles into PMMA substrate through PMMA mask and plasma etching, and chemically modifying the unprotected particle surface. The strategy of preparing Janus particles through Pickering emulsion template method is to disperse the modified particles at the oil/water interface, immerse part of the particles into the oil phase, and the other part is in the water phase. When the particles are reacted in the oil phase or/or water phase, the surface of the particles will be modified, so that Janus particles with different structure and/or chemical composition can be prepared (Pardhy and Budhlall, 2010). Tanaka et al. (2010) dissolved the incompatible polymethylmethacrylate (PMMA) and polystyrene (PS) in toluene, then dispersed the toluene droplets in the surfactant aqueous solution, evaporated the solvent after emulsification, and separated PMMA and PS, so as to prepare Snowman shaped Janus particles. Deng et al. (2015) prepared chloroform solution of polystyrene poly four vinyl pyridine (PS-P4VP) two block copolymer as oil phase and aqueous solution of polyvinyl alcohol as water phase to prepare O/W emulsion. At the interface, polystyrene/poly (four vinyl pyridine) hetero shell particles were formed, and then PS/P4VP Janus particles were redispersed through the selective crosslinking of poly four vinyl pyridine.

In order to combine the advantages of hydrophilic particles in reducing capillary resistance and the advantages of detaining oil by decompression and injection of hydrophobic particles (Ramsden, 1904; Fang et al., 2019). The amphiphilic asymmetric Janus nanoparticles were prepared to optimize injection effect and enhance oil and gas recovery. Amphiphilic nanoparticles can effectively exert their advantages in physical and chemical properties in oil-water interface and form close sequence at oil-water interface. Compared with low molecular weight surfactant and single modified nanoparticle stabilized emulsion, asymmetric Janus nanoparticles can effectively stabilize emulsion and achieve dynamic equilibrium.

Nano-flooding agent is based on small size, so the larger the specific surface area, the larger the area of functional groups in the process of oil displacement, and the stronger the ability of adsorbing and desorbing crude oil (Synytska et al., 2011; Sun et al., 2014). In this paper, asymmetric Janus nanoparticles were synthesized by covalent coupling modification. The round particles were transformed into nanosheets by mechanical shearing. The physical and chemical properties and oil displacement efficiency of circular and square Janus nano-flooding agents were compared and analyzed.

## Materials and Methods

### Material

Nano-silica particles (diameter 10 nm) were purchased from Shanghai Keyan Industrial Co., Ltd. (Shanghai, China). Fully refined paraffin particles (melting temperature range: 58°C) were purchased from Beijing Haibeisi Company (Beijing, China). Gamma-methacryloxypropyl trimethoxysilane (gamma-MPS) (99%) was purchased from Dongguan Shanyi Plasticizing Co., Ltd. (Dongguan, China), acetone, cetyltrimethylammonium bromide (CTAB) from Tianjin Guangfu Fine Chemical Research Institute (Tianjin, China). Ferrous sulfate heptahydrate, ferric sulfate and anhydrous ethanol were purchased from Guangzhou Chemical Reagent Factory (Guangzhou, China), ammonia water and Guangzhong Guanghua Chemical Factory Co., Ltd. (Guangzhou, China). Simulated crude oil, a production plant in Daqing Oilfield is compounded with kerosene with a viscosity of 9.8mPa.s at 45°C.

### Preparation of Amphiphilic Asymmetric Janus Nanosheets and Characterization

**Figure 1:**
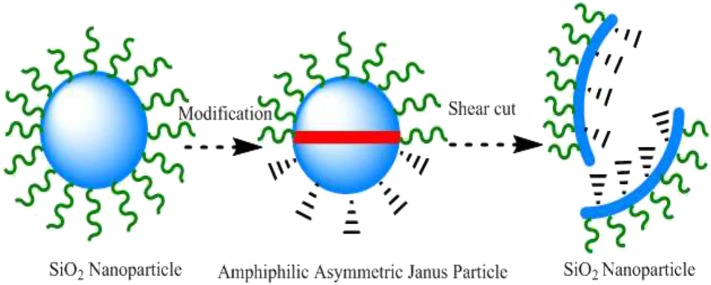
Preparation of amphiphilic asymmetric Janus nanosheets.

The amphiphilic affinity asymmetric Janus nanosheets were synthesized by Pickering emulsion (Ruhland et al., 2013), and the synthesis scheme of asymmetric two affinity nanoparticles was shown in Figure 1. The particles and solid paraffins were dispersed in ultrasonic water with molar mass of 1:3, and emulsified for 2 h under constant temperature stirring of 1,500 r/min. Immediately after the emulsification, the emulsion was placed in the refrigerator freezer and cooled to the solid state of particles. The solid surface was washed with supernatant water for three times and then dried in vacuum at 45°C. The dried particles were dispersed in 0.1% gamma-MPS ethanol solution and reacted at room temperature for 72 h. After the reaction, the particles were filtered and washed with ethanol, then soaked in trichloromethane and centrifugally rinsed. Then half of the syntheses were prepared into nanosheets by mechanical shearing (shear rate 14,000 rpm and shear time 10 min). It was dried in a vacuum drying box for 24 h for later use.

The shear force of high shear disperser is much greater than that of ordinary shear disperser. The high-speed shear disperser makes the materials collide with each other through the high-speed rotation of the blade shaft. Under the action of these forces, nanoparticles are broken rapidly to be nanosheets. This makes the dispersing effect of nanosheets greatly enhanced.

The surface morphology of Janus nanosheets was observed by scanning electron microscopy (SEM, JEOL Co., Tokyo, Japan). The chemical modification of Janus nanosheets was detected by WQF-410 Fourier Transform Infrared Spectrometer (FTIR) of Malvern Company, UK. The particle size distribution of nanosheets was measured by Mastersizer 3000 laser particle size analyzer of Malvern Company, UK. By using SQUID MPMS XL-7 magnetometer, Tristan Company, USA, under 298 K experimental conditions, the specific saturation magnetization (Ms) of particles was obtained by using the maximum magnetic field of 1 × 10^5^e.

### Preparation of Fe_3_O_4_@SiO_2_ Paramagnetic Amphiphilic Janus Nanosheets

The preparation of paramagnetic nanosheets is conducive to recovery and reuse after displacement. Fe_3_O_4_@SiO_2_ amphiphilic asymmetric Janus nanosheets were prepared by coprecipitation method (De Gennes, 1992; Liu et al., 2008; Du and Reilly, 2011). Two reagents, ferrous sulfate heptahydrate and ferric sulfate solution, were mixed in a molar ratio of 1:2 and quickly put into 100 ml ammonia aqueous solution. Argon was introduced and stirred continuously for 1 h before magnetic separation.

### Interfacial Activity of Amphiphilic Asymmetric Janus Nanosheets

In order to analyze the interfacial activity of amphiphilic asymmetric Janus nanosheets, TX500C interfacial tension tester was used to characterize the interfacial activity. Janus nanosheets with different concentration gradients (0.01, 0.05, 0.1, 0.15, and 0.2 wt%) were set to determine the interfacial tension values in water with different salinities (distilled water, 4,000, 6,000, 8,000, 12,000, and 20,000 mg/l). The optimum concentration and salt tolerance of nanosheets were determined by determination.

### Emulsifying Properties of Amphiphilic Asymmetric Janus Nanosheets

The mechanism study of enhancing oil and gas recovery shows that it is inappropriate to use interfacial tension method alone to evaluate displacement formula. Therefore, the emulsion droplet coalescence rate and the stability of the emulsion will also affect the displacement efficiency, and then carry out the emulsification experiment. The volume ratio of 0.1 wt% Janus nanosheet dispersion solution to simulated crude oil was 1:1, and 10 ml of Janus nanosheet dispersion solution was placed in 25 ml colorimetric flask, respectively. The mixture was well-mixed by hand for 5 min, 40 times/min, and placed at 45, 65, and 85 degrees, respectively. Observation of emulsification dynamics, calculation of dehydration rate and analysis of emulsification stability of nanosheets.

### Calculation of Resistance Coefficient and Oil Displacement Efficiency

Reaction materials and conditions: Core parameters are shown in Tables 1, 2. The injection rate is 0.2 ml/min, the experimental temperature is 45°C, and the water used in the experiment is tap water. The water permeability of the core used in the experiment is 10 × 10^−3^ and 50 × 10^−3^ μm^2^, respectively.

**Table 1:** 10 × 10^−3^ μm^2^ Core parameters.

**Parameters**	**Core type**	
	**Low permeability core L1**	**Low permeability core L2**
Core length (cm)	9.80	9.81
Core diameter (cm)	2.5	2.5
Water permeability measurements (10^−3^μm^2^)	9.6	10.2
Pore volume (cm^3^)	5.2	5.3
Porosity (%)	10.8	11.0

**Table 2:** 50 × 10^−3^ μm^2^ Core parameters.

**Parameters**	**Core type**	
	**Low permeability core L3**	**Low permeability core L4**
Core length (cm)	9.82	9.80
Core diameter (cm)	2.5	2.5
Water permeability measurements (10^−3^μm^2^)	55	52
Pore volume (cm^3^)	6.5	6.4
Porosity (%)	13.5	13.3

**Figure 2:**
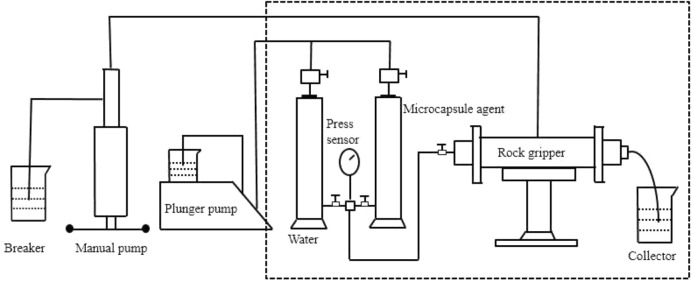
Experimental device diagram.

Specific experimental steps (The experimental device is shown in Figure 2.

1. The core is vacuumed for 4 h and saturated with 6,000 mg/l mineralized water. The porosity and permeability are calculated and the pressure PW is recorded.
2. The core used in the experiment of emulsion connection is used to study the seepage law of the emulsified system.
3. To calculate the drag coefficient by selecting the average value of the increasing pressure of the injected emulsion, the resistance coefficient of R_f_ = P_W_/P_E_ is used to characterize the influence of different factors in porous media on the emulsifying capacity of the system. Among them: the pressure of core permeability measured by P_W_-water, the corresponding value of the stable section of pressure curve after P_E_-injection of emulsion, and the resistance coefficient of R_f_-emulsion.

## Results

### Characterization of Amphiphilic Asymmetric Janus Nanosheets

#### Fourier Transform Infrared Spectroscopy

The infrared spectra of nano-silica and silica @ modifier were determined, respectively. Comparing the changes of characteristic absorption peaks of silica before and after modification, the main structural characteristics of modified silica surface were qualitatively analyzed.

**Figure 3:**
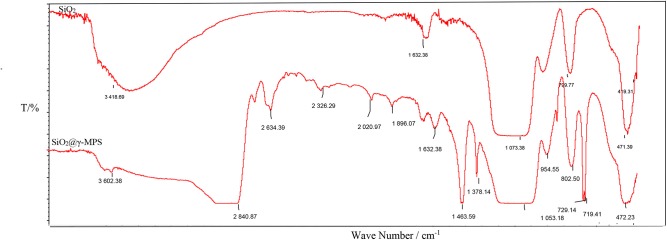
Comparison of infrared spectra before and after nano-silica modification.

As shown in Figure 3, nano-silica has four characteristic absorption peaks, namely - OH, 3,418.69 cm^−1^; H-O-H, 1,632.38 cm^−1^; Si-OH, 1,073.38 cm^−1^; Si-O, 471.39 cm^−1^, 419.31 cm^−1^. After functional modification, the -OH on the surface of silica crosslinked with the modifier to form a new chemical bond. By adjusting the reaction amount of modifier, different surface area films can be formed on the surface of silica. By comparing the infrared spectra before and after the reaction, we can find that there are many -CH_3_/-CH_2_ vibration peaks and Si-O-C absorption peaks, which prove that the modifier can make the silicon dioxide surface organic.

#### Magnetization Measurement

**Figure 4:**
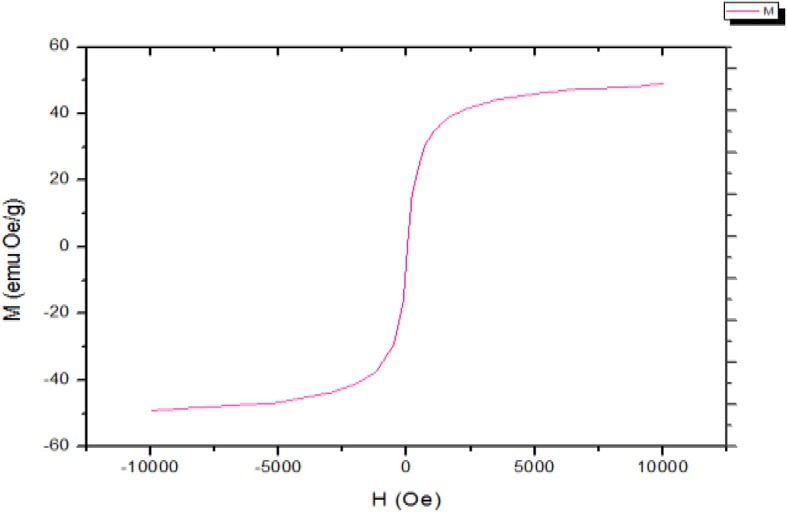
Magnetization force curves for Fe_3_O_4_@SiO_2_ amphiphilic asymmetric Janus nanosheets (25°C).

In order to recover Janus nanosheets effectively in the later stage of the experiment, paramagnetic Fe_3_O_4_@SiO_2_ nanosheets were prepared. The hysteresis loop of the sample was obtained by using a magnetometer at 298 K and using a maximum magnetic field of 1 × 10^5^e (Xu et al., 2001; Liu et al., 2008; Liang et al., 2011; Walther and Müller, 2013). Figure 4 shows the magnetic force curve (25°C) of the asymmetric Janus nanosheets with Fe_3_O_4_@SiO_2_ amphiphiles. Through chemical conversion, the specific saturation magnetization of the particles is 49.0 emu/g. As can be seen in the figure, only when a magnetic field is applied, the magnetization distribution will be aligned in a directional direction and the field withdrawal alignment will disappear. Therefore, the prepared Fe_3_O_4_@SiO_2_ nanosheets have superparamagnetism.

#### Dimension Analysis

**Figure 5:**
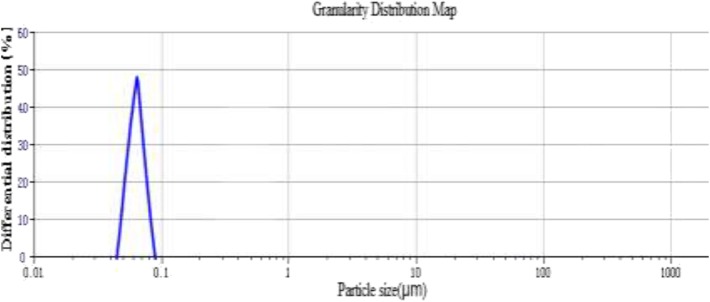
Size distribution of Janus nanoparticles.

Malvern laser particle size analyzer was used to measure distilled water as dispersing medium. The results show that the median diameter D50 of Janus nanoparticles is 66.70 nm and the specific surface area is 18563.802 m^2^/kg. As shown in Figure 5, Janus nanoparticles are well-dispersed and uniformly distributed.

#### Electron Microscopic Analysis

**Figure 6:**
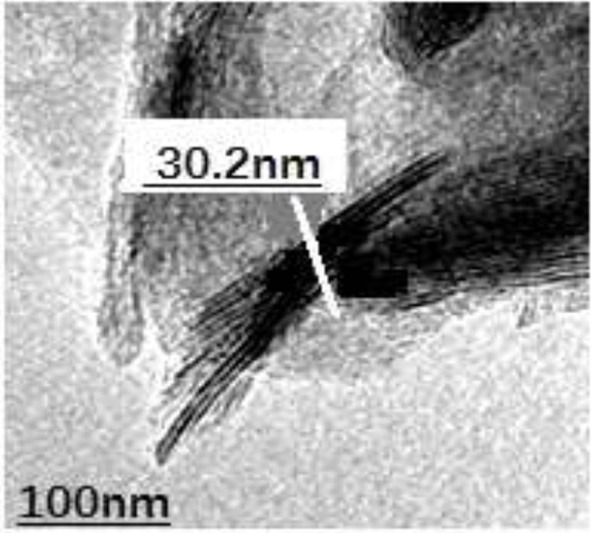
Electron microscopy of nanosheets.

As shown in Figure 6, the nanosheet was successfully prepared by the observation of the electron microscopy of the nanosheet. The thickness of the nanosheet is about 45% of the nanoparticles.

### Interfacial Activity of Nanosheets

#### Salt Resistance Assessment

The experimental oil is composed of crude oil and kerosene from a factory in Daqing, and its viscosity is 9.8 mPa·s at 45°C. The interfacial tension of nano-tablets is measured by spin-drop method at 45°C. The Janus nanosheets shown in Table 3 have good salt tolerance. The interfacial tension of Janus nanosheets with different concentration in different mineralized water is 10^−2^ mN.m^−1^, and the salt tolerance is strong. The interfacial tension of 0.1 Wt% nanosheets in high salinity water can reach 10^−3^ m N· m^−1^, and that of 0.15 and 0.2 wt% nanosheets in high salinity water can reach 10^−4^ m N· m^−1^. The ultra-low interfacial tension is due to the salinity condition in the given range. The active molecules of the nanosheets form micelles, and the electrolyte enrichment makes the nanosheets closely arranged on the interface, making the oil-water interface reach ultra-low.

**Table 3:** Statistical table of salt resistance of Janus nanosheets.

**Interfacial tension /mN·m**^**−1**^ **(temperature 45**^**°**^**C)**		**Salinity /mg·l**^**−1**^				
		**4,000**	**6,000**	**8,000**	**12,000**	**20,000**
concentration/wt%	0.01	3.2 × 10^−1^	6.8 × 10^−2^	3.7 × 10^−2^	1.9 × 10^−2^	1.3 × 10^−2^
	0.05	7.7 × 10^−2^	2.5 × 10^−2^	6.9 × 10^−3^	5.1 × 10^−3^	3.5 × 10^−3^
	0.1	8.2 × 10^−3^	5.1 × 10^−3^	4.2 × 10^−3^	2.2 × 10^−3^	9.4 × 10^−4^
	0.15	5.3 × 10^−3^	3.9 × 10^−3^	8.1 × 10^−4^	7.1 × 10^−4^	6.9 × 10^−4^
	0.2	4.3 × 10^−3^	2.7 × 10^−3^	7.5 × 10^−4^	5.3 × 10^−4^	4.5 × 10^−4^

#### Evaluation of Temperature Resistance

As shown in Table 4, the interfacial tension of 0.05–0.2 wt% nanosheet dispersions can reach 10^−2^ m N· m^−1^ at 45 to 80°C. The interfacial tension of 0.15 and 0.2 wt% nanosheets can reach 10^−3^ m N· m^−1^ at 60°C.

**Table 4:** Temperature resistance of Janus nanosheets.

**Interfacial tension /mN·m**^**−1**^ **(Salinity 4000 mg·L**^**−1**^**)**		**Temperature/**^**°**^**C**		
		**45**	**60**	**80**
concentration/wt%	0.01	3.2 × 10^−1^	6.5 × 10^−1^	8.7 × 10^−1^
	0.05	7.7 × 10^−2^	8.5 × 10^−2^	9.1 × 10^−2^
	0.1	8.2 × 10^−3^	4.6 × 10^−2^	4.2 × 10^−2^
	0.15	5.3 × 10^−3^	8.6 × 10^−3^	3.1 × 10^−2^
	0.2	4.3 × 10^−3^	7.2 × 10^−3^	2.7 × 10^−2^

### Emulsifying Property

Five concentrations of nanosheet dispersions (4,000 mg/L^−1^ mineralized water) of 0.01, 0.05, 0.10, 0.15, and 0.2 wt% were mixed with simulated crude oil in a volume ratio of 1:1, respectively, and placed in a 25 ml colorimetric tube. The speed of magnetic stirrer is 300 r/min at 45°C, and the stirring time is 5 min (the bubbles produced by rapid stirring cause the instability of emulsion). The thermostat at 45°C is stationary.

**Figure 7:**
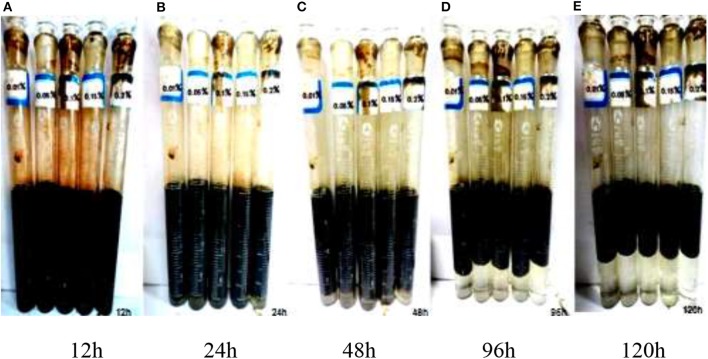
Emulsification 120 h photos. **(A)** 12 h. **(B)** 24 h. **(C)** 48 h. **(D)** 96 h. **(E)**120 h.

The results show as shown in Figure 7 that the dehydration rate of 120 h nanosheet dispersions is 100%. After 48 h, the dehydration rate of nanosheet dispersions is nearly 5%. After 96 h, the dehydration rate of nanosheet dispersion is nearly 25%. Therefore, nanosheets have good emulsifying ability.

It can be seen from the above experiments that Janus smart nanosheet has good emulsification stability. In order to further determine its emulsification ability, the resistance coefficient of the nanosheet after emulsification is determined. The original oil saturation of the experimental cores is about 70%. 0.01 wt% nanosheets were displaced after water flooding. As shown in the Table 5, the resistance coefficient after emulsification is obtained by the pressure change before and after displacement.

**Table 5:** Data sheet of resistance coefficient of nanosheet drive after water flooding.

**Chemical agent**	**Permeability (10**^**−3**^**μm**^**2**^**)**	**Resistance coefficient**
0.01% Janus smart nanosheet	10.2	25.3
	55	14.2

**Figure 8:**
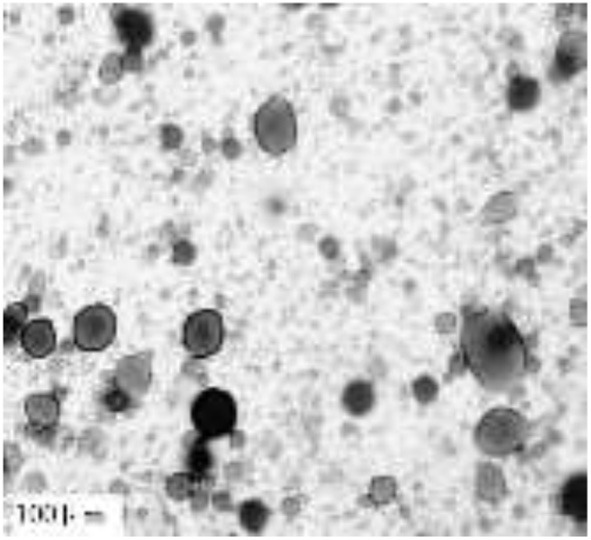
Nanomaterial emulsion microscope photograph.

As shown in Figure 8, the emulsion droplets of nanosheets can penetrate oil into the sheet structure, dismantle the overlapping stacked aggregates, break up their continuous state, effectively control the geometric size and stacking between them, and improve the stability time of emulsification.

### The Interaction Between Smart Nanosheets and Polymers

**Figure 9:**
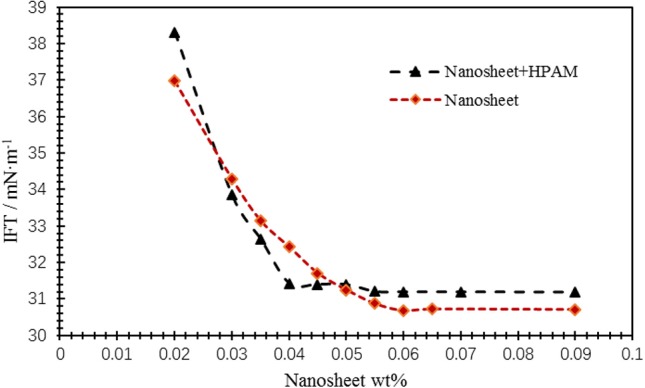
Interaction diagram of smart nanosheets and polymers.

Smart nanosheet is a kind of surfactant with amphiphilic properties, which is obtained by modifying nano silica with coupling agent and cationic polyelectrolyte. The nanosheet has the same beauty as the surfactant for oil displacement. They all have hydrophilic and lipophilic groups. The difference is that a nano particle or a nanosheet is a collection of many surfactant molecules. It has multiple hydrophilic and lipophilic groups at the same time, which greatly enhances the interfacial activity of the nano body. Compared with the conventional surfactants, the modified nano-sized materials have better adsorption resistance. In reservoir development, polymer and surfactant are often used in combination. Therefore, the surface tension of single component and partially hydrolyzed polyacrylamide can be determined to determine whether there is competitive adsorption between them. As shown in Figure 9, when the concentration of the nanosheet dispersion is lower than its CMC value, the surface tension decreases after adding the polymer, and when it is higher than the CMC value, the interfacial tension of the polymer increases slightly (the polymer concentration is 100 mg/L). It is said that with the critical micelle concentration as the boundary, the surface tension can be reduced when the concentration is lower than that of the nanosheet and the polymer; when the concentration is higher than that of the nanosheet, the molecular number of the nanosheet will be reduced and the surface tension will be increased slightly, but it is still at a lower value. It can be seen from the data distribution that the addition of polymer reduces the critical micelle concentration of the system, and can reduce the amount of nanosheet to a certain extent. Therefore, the cooperation between the polymer and the nanosheet is the main way. In the formulation design, the concentration of nanosheet is increased, the competitive adsorption cannot be considered.

### Displacement Efficiency

The data obtained from oil displacement experiments are shown in Table 6. Experiment showed that nanosheets could increase oil and gas recovery by 7.80%.

**Table 6:** Displacement of nanosheets.

**System formula**	**Interfacial tension (mN/m)**	**Water flooding recovery factor (%)**	**EOR enhanced value (%)**	**Total recovery factor (%)**
0.15% nanosheets	5.3 × 10^−3^	37.41	7.80	45.21
0.15% nanosheets + 0.1%HPAM	5.1 × 10^−3^	37.55	17.20	54.75

**Figure 10:**
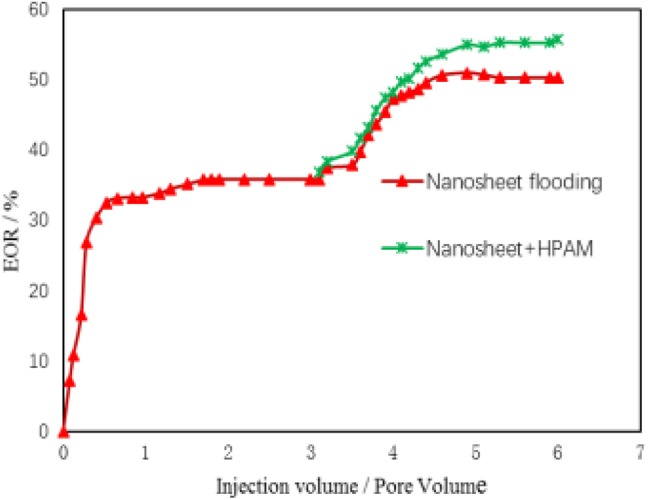
Displacement effect curve.

As shown in Figure 10, the combination of nano tablets and polyacrylamide can further improve oil and gas recovery. At the same time, the addition of the polymer did not affect the interfacial activity of the nanosheets.

**Figure 11:**
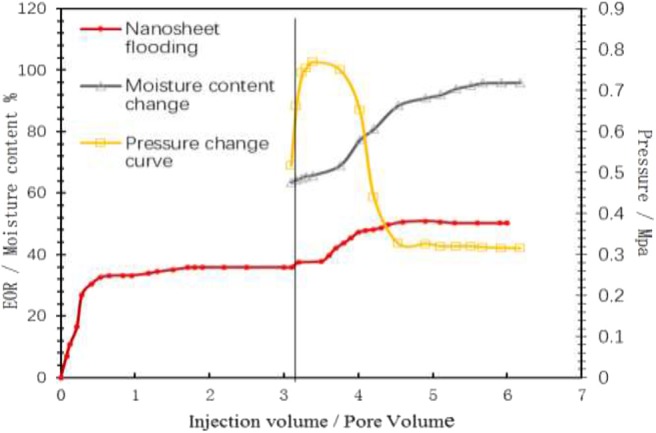
Oil displacement dynamic curve of nanosheet.

As shown in Figure 11, the curves of water cut, oil recovery and displacement pressure with injection volume are shown. It can be seen from the experimental data in the table that the oil recovery can be increased by 7.8% and the final oil recovery can reach 45.21% based on water flooding. After injection into the core, spontaneous emulsification occurs in the core as time goes on. From the change of displacement pressure, it can be seen that the subsequent injection water resistance increases significantly. The results show that nanosheet can start residual oil in core, expand sweep volume and enhance oil recovery.

### Analysis of Particle Regulation

Janus nanoparticles need to adjust the amount of modifier to determine the degree of particle coating in order to maintain good hydrophilic and hydrophilic properties of the sphere. The surface free energy was calculated by measuring the contact angle of silica particles before and after modification, and the change degree of surface free energy was determined by the ratio of modifiers. The surface free energy of silica before modification is 72 mJ/m^2^, and that of solid paraffin is 32 mJ/m^2^. Therefore, when the coating rate is 0, the surface free energy of Janus nanoparticles is 72 mJ/m^2^, and that of Janus nanoparticles is 32 mJ/m^2^ when the coating rate is 100%. When the corresponding grafting area is 0, the contact angle is 0, the grafting area is 50% (i.e., uniform modification), the contact angle is 90 and the grafting area is 100%, the oil-wet and the contact angle is 180. Therefore, suitable coating rate plays an important role in the wettability of nanoparticles, so it is necessary to quantify the grafting density (Perro et al., 2005, 2009; Schmitt-Rozières et al., 2009; Lee et al., 2010).

**Figure 12:**
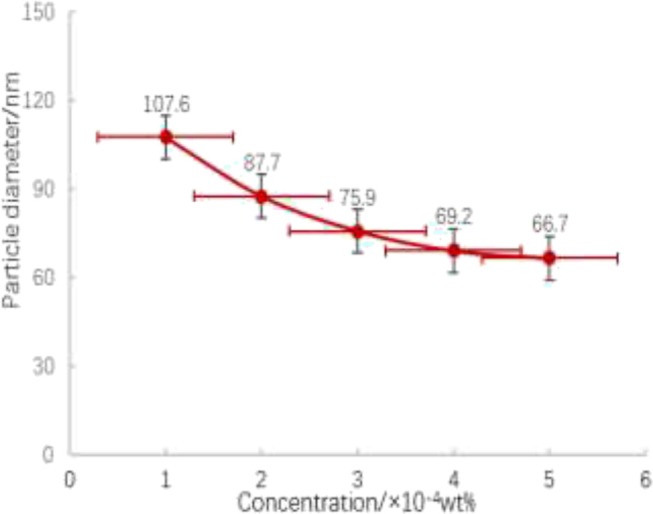
Gradient diagram of Janus nanoparticle size with CTAB concentration.

The formula of graft density was defined as $\varpi \text{=}\frac{\text{d}\rho {\text{N}}_{\text{A}}}{M}$, ϖ as graft density nm^−2^, d as particle diameter nm^3^, graft monomer density 1 g/cm^3^, N_A_ as Avogadro constant, *M* as relative molecular weight of graft monomer. The spacing of grafted monomers was expressed in $\iota {=}^{1}{/}_{\sqrt{\varpi }}$. The mass ratio of nano-silica to paraffin wax is 1:7. The shape and size of particles can be optimized by adjusting the amount of CTAB. The nanoparticle size was determined by comparing the dosage of 1, 2, 3, 4, and 5 mg/l CTAB. As shown in Figure 12, the particle size is the smallest under the conditions of 4 and 5 mg/l CTAB. Taking 66.7 nm nanoparticles as an example, according to the formula, the grafting density is 1.72 nm^−2^, the distance between grafting monomers is 1.31 nm, the grafting area is 38.8%, the contact angle is 70 degrees, and the surface free energy is 46.9 m J/m^2^.

## Conclusion

The purpose of this study is to prepare amphiphilic asymmetric Janus nanoparticles with interfacial activity by using silica nanoparticles. The size of nanoparticles can be controlled by adjusting the proportion of modifiers. The nanosheets with larger specific surface area and smaller particle size were formed by shearing process. The nanomaterial has outstanding interfacial activity and good temperature and salt resistance. At low concentration, the material can achieve ultra-low interfacial tension of 10^−4^ mN/m. The prepared Fe_3_O_4_@SiO_2_ nanosheets have superparamagnetism, which provides an effective means for displacement recovery. Controllable assembly and application of asymmetric Janus smart nanosheets for oil displacement provide new research ideas for the development of nano-displacement agents, which have certain reference value.

The main results are as follows: (1) Janus smart nanosheets of given size were obtained by surface modification and high shear stress shearing. (2) Fe_3_O_4_@SiO_2_ Magnetic Janus nanocomposites were prepared by coprecipitation. The preparation of magnetic nanoparticles is conducive to the subsequent mechanism analysis of displacement of nanoparticles, and is also conducive to recycling. (3) At the salinity of 4,000 mg /l, the interface tension of 0.15–0.2 wt% nanosheets is very low at 45–80°C. When the salinity is increased to 20,000 mg /l, it is found that the interfacial tension decreases continuously, even reaches 10^−4^ mN/m at a certain temperature and salinity. (4) The results of emulsification experiment show that the rate of water evolution reaches 100% after 120 h. (5) Nanosheets have better emulsification ability, nanosheets emulsion has a certain resistance coefficient, which can enhance the recovery degree in the displacement experiment. The recovery degree of 0.15 wt% nanosheets displacement experiment is 7.8%. (6) In the process of displacement, the competitive adsorption of nanosheets and polymers does not affect the interfacial activity of nanosheets, and the combination of the two can effectively improve the displacement efficiency, 0.15% nanosheets+0.1% HPAM enhanced oil recovery by 17.2%. (7) Through theoretical calculation, we can adjust the proportion of the formula of the nanosheets and effectively prepare the nanosheet with the given particle size.

## Data Availability Statement

All datasets generated for this study are included in the article/supplementary material.

## Author Contributions

FS and JW: conceptualization and formal analysis. FS: investigation, methodology, validation and writing—original draft preparation. JW: writing—review and editing. BZ and YZ: data curation and visualization. XK: proofreading and revision of manuscript.

### Conflict of Interest

BZ and XK were employed by the company PetroChina Daqing Oilfield Co., Ltd. The remaining authors declare that the research was conducted in the absence of any commercial or financial relationships that could be construed as a potential conflict of interest.

## References

[B1] Aveyard R. Binks B. P. Clint J. H. (2003). Emulsions stabilised solely by colloidal particles. Adv. Colloid Interface Sci. 100, 503–546. 10.1016/S0001-8686(02)00069-6

[B2] Binks B. P. Clint J. H. (2002). Solid wettability from surface energy components: Relevance to Pickering emulsions. Langmuir 18, 1270–1273. 10.1021/la011420k

[B3] De Gennes P. G. (1992). Soft matter. Rev. Mod. Phys. 64:645 10.1103/RevModPhys.64.645

[B4] Deng R. Liang F. Qu X. Wang Q. Zhu J. Yang Z. (2015). Diblock copolymer based Janus nanoparticles. Macromolecules 48, 750–755. 10.1021/ma502339s

[B5] Du J. Reilly R. K. (2011). Anisotropic particles with patchy, multicompartment and Janus architectures: Preparation and application. Chem. Soc. Rev. 40, 2402–2416. 10.1039/c0cs00216j 21384028

[B6] Fang S. Wu J. Zhao B. (2019). Preparation and investigation of intelligent polymeric nanocapsule for enhanced oil recovery. Materials 12:1093 10.3390/ma12071093 PMC6479461 30987019

[B7] Finkle P. Draper H. D. Hildebrand J. H. (1923). The theory of emulsification. J. Am. Chem. Soc. 45, 2780–2788. 10.1021/ja01665a002

[B8] Glaser N. Adams D. J. Böker A. Krausch G. (2006). Janus particles at liquid-liquid interfaces. Langmuir 22, 5227–5229. 10.1021/la060693i 16732643

[B9] Lee Y. Garcia M. A. Frey Huls N. A. Sun S. (2010). Synthetic tuning of the catalyticproperties of Au-Fe3O4 nanoparticles. Angew. Chem. Int. Ed. 122, 1293–1296. 10.1002/ange.200906130 20077449

[B10] Liang F. Liu J. Zhang C. Qu X. Li J. Yang Z. (2011). Janus hollow spheres by emulsion interfacial self-assembled sol-gel process. Chem. Commun. 47, 1231–1233. 10.1039/C0CC03599H 21103498

[B11] Ling X. Y. Phang I. Y. Acikgoz C. Yilmaz M. D. Hempenius M. A. Vancso G. J.. (2009). Janus particles with controllable patchiness and their chemical functionalization and supramolecular assembly. Angew. Chem. Int. Ed. 48, 7677–7682. 10.1002/anie.200903579 19746380

[B12] Liu B. Wei W. Qu X. Yang Z. (2008). Janus colloids formed by biphasic grafting at a Pickering emulsion interface. Angew. Chem. Int. Ed. 120, 4037–4039. 10.1002/ange.200705103 18421735

[B13] Pardhy N. P. Budhlall B. M. (2010). Pickering emulsion as a template to synthesize Janus colloids with anisotropy in the surface potential. Langmuir 26, 13130–13141. 10.1021/la101502e 20695551

[B14] Perro A. Meunier F. Schmitt V. Ravaine S. (2009). Production of large quantities of Janusnanoparticles using wax-in-water emulsions. Colloids Surf. A Physicochem. Eng. Asp. 332, 57–62. 10.1016/j.colsurfa.2008.08.027

[B15] Perro A. Reculusa S. Ravaine S. Bourgeat-Lami E. Duguet E. (2005). Design and synthesis of Janus micro-and nanoparticles. J. Mater. Chem. 15, 3745–3760. 10.1039/b505099e

[B16] Ramsden W. (1904). Separation of solids in the surface-layers of solutions and suspensions. Proc. R. Soc. Lond. 72, 156–164. 10.1098/rspl.1903.0034

[B17] Roh K. H. Martin D. C. Lahann J. (2005). Biphasic Janus particles with nanoscale anisotropy. Nat. Mater. 4, 759–763. 10.1038/nmat1486 16184172

[B18] Ruhland T. M. Gröschel A. H. Ballard N. Skelhon T. S. Walther A. Müller A. H.. (2013). Influence of Janus particle shape on their interfacial behavior at liquid–liquid interfaces. Langmuir 29, 1388–1394. 10.1021/la3048642 23311383

[B19] Schmitt-Rozières M. Krägel J. Grigoriev D. O. Liggieri L. Miller R. Vincent-Bonnieu S.. (2009). From spherical to polymorphous dispersed phase transition in water/oil emulsions. Langmuir 25, 4266–4270. 10.1021/la804214m 19281158

[B20] Sun Q. Li Z. Li S. Jiang L. Wang J. Wang P. (2014). Utilization of surfactant-stabilized foam for enhanced oil recovery by adding nanoparticles. Energy Fuels 28, 2384–2394. 10.1021/ef402453b

[B21] Synytska A. Khanum R. Ionov L. Cherif C. Bellmann C. (2011). Water-repellent textile via decorating fibers with amphiphilic janus particles. ACS Appl. Mater. Interfaces 3, 1216–1220. 10.1021/am200033u 21366338

[B22] Tanaka T. Okayama M. Minami H. Okubo M. (2010). Dual stimuli-responsive mushroom-like Janus polymer particles as particulate surfactants. Langmuir 26, 11732–11736. 10.1021/la101237c 20507141

[B23] Walther A. Müller A. H. E. (2013). Janus particles: synthesis, self-assembly, physicalproperties, and applications. Chem. Rev. 113, 5194–5261. 10.1021/cr300089t 23557169

[B24] Xu H. Erhardt R. Abetz V. Müller A. H. Goedel W. A. (2001). Janus micelles at the air/water interface. Langmuir 17, 6787–6793. 10.1021/la010091t

